# Early-Age Properties of Concrete Based on Numerical Hydration Modelling: A Parametric Analysis

**DOI:** 10.3390/ma13092112

**Published:** 2020-05-02

**Authors:** Marco Pepe, Carmine Lima, Enzo Martinelli

**Affiliations:** 1 Department of Civil Engineering, University of Salerno, Via Giovanni Paolo II 132, 84084 Fisciano, SA, Italy; mapepe@unisa.it (M.P.); c.lima@tesis-srl.eu (C.L.); 2 TESIS srl, Via Giovanni Paolo II 132, 84084 Fisciano, SA, Italy

**Keywords:** hydration modelling, thermal cracking, early-age mechanical properties

## Abstract

The early-age performances of cement-based mixtures are governed by cement hydration reactions. As a matter of fact, the heat generated during the setting and hardening phases due to the hydration processes increases the temperatures within the concrete elements while it starts developing its mechanical properties. These thermal stresses can cause the premature cracking of the cementitious matrix and undermine the long-term durability of the whole concrete element, especially in the case of massive structures where the dissipation of generated heat is more difficult. It is worth highlighting that the kinetics of cement hydration is mainly governed by the mixture composition; on the other hand, the heat generated during the setting and hardening is also influenced by the geometry of the element and/or its curing conditions. In this context, this study presents a numerical procedure intended to simulate the hydration reactions, and hence scrutinize the development of concrete properties at the early-age. Specifically, considering the variation of several factors, such as concrete strength class, element size and curing conditions, a comprehensive parametric analysis is presented herein, leading to the proposal of a simplified approach for both predicting the time evolution of the concrete mechanical performances at the early-age and mitigating the risk of premature cracking.

## 1. Introduction

The heat generated during the setting and hardening phases of cement hydration increases the temperature within the concrete elements and, consequently, it can result in cracking phenomena that compromise the long-term durability performance of concrete structures [1]. Generally, the cracking risk is considered relevant only in massive structures, where the dissipation of the generated heat is relatively slow, and therefore high temperature gradients take place inside the concrete elements [2,3]. It is worth highlighting that the heat generated during the hydration reaction depends on various relevant factors, such as the physical-chemical property of the mixture (e.g., type of binders, quality of aggregates and water-to-cement ratio), the geometry of the elements and the boundary conditions (e.g., ambient temperature and type of formwork) [4,5].

Therefore, nowadays, with the diffusion of high-performance cementitious composites, the cracking risk in concrete at the early-age is no longer limited to the case of massive structures [6,7,8]. As a matter of fact, the higher amount of employed binders associated with lower water-to-cement ratios lead to a higher heat of hydration and finer pores characterizing the corresponding cement microstructure, which, in turn, generates both a wider thermal gradient and autogenous shrinkage [9,10].

On the other hand, the setting and hardening processes developing in cement-based mixtures can be monitored by analyzing the hydration kinetics which can be scrutinized by the so-called degree of hydration and, at the same time, the latter can be correlated to the development of the relevant physical and mechanical performance of the resulting composites [11,12].

In principle, the degree of hydration, which is measured by the heat generated in the hydration reaction, can be easily determined only in the case of reactions developing under adiabatic conditions, as, in that case, the generated heat is proportional to the temperature development within the concrete mixtures [13]. Conversely, under more general boundary conditions (e.g., semi-adiabatic or isothermal), advanced techniques, such as scanning electron microscopy [14], nuclear magnetic resonance [15] and/or X-ray diffraction (XRD) analysis [16], are required to determine the time evolution of the degree of hydration in curing concrete.

In this context, the present work firstly summarizes a numerical procedure that is capable of simulating the time evolution of the early-age temperature development also cured in non-adiabatic conditions: this modelling/identification procedure can be used to simulate the time evolution of the degree of hydration of a concrete by considering various boundary conditions [17].

The implementation of the proposed modelling approach allows to implement an “implicit” verification procedure for cracking phenomena occurring during the hydration processes. Moreover, since the degree of hydration represents the evolution of the microstructure formation, it can be correlated to the development of the associated mechanical properties, such as the compressive/tensile strength and the elastic modulus [18,19].

Based on these assumptions, a parametric analysis is proposed by considering the variation of the following parameters: concrete mix design, structural element geometry and boundary conditions (i.e., type of formwork and ambient temperature). In principle, these parameters lead to control and prevent the cracking risk by optimizing the following aspects: cement-based mixture composition (i.e., type and temperature of the compounds), casting procedure and the use of heat-insulating formwork for the entire duration of the casting maturation.

## 2. Modelling Approach

### 2.1. Hydration Processes

As a matter of principle, the development of the hydration reactions in cement-based mixtures represent a complex problem in concrete technology since the hydration degree of individual phases (i.e., C_3_S, C_2_S, C_3_A and C_4_AF) in Portland cement present different kinetics. For these reasons, it is a common practice to also define the degree of hydration (α_h_) as a general indicator (i.e., not considering the differences among the several phases) of the hydration processes occurring in concrete mixtures during setting and hardening [13].

On the other hand, as it is well known, the time evolution of the degree of hydration α_h_(t) can be also defined as the ratio between the amount of heat Q(t) produced up to the time t, and the heat Q_max_ potentially produced by the reaction of the total amount of cement [17]:

(1) $$\alpha_{h}\left(t\right)=\frac{Q\left(t\right)}{Q_{\max}}$$

**Figure 1:**
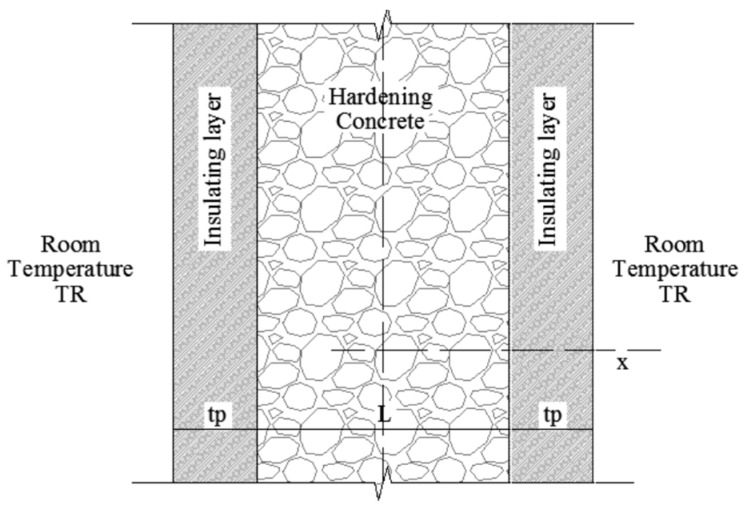
Geometrical description of the 1-D problem (adapted from [19]).

Consequently, the cement hydration results in a heat transfer process and then a transient temperature field T. It is worth mentioning that, in most of the relevant cases of massive concrete structures (i.e., dams, foundation slabs, etc.) [2], the temperature field can be schematized as a 1-D domain as highlighted in Figure 1, in which the heat flow is predominant in one direction.

The T field developing in a 1-D domain can be described by the following equation:

(2) $$\rho_{c}c_{c}\frac{\partial T}{\partial t}=\lambda_{c}\cdot \frac{\partial^{2}T}{\partial x^{2}}+q_{c}(x,t)$$

where ρ_c_ is mass per unit volume, c_c_ is the specific heat, λ_c_ is the thermal conductivity coefficient and q_c_(x,t) is the rate of heat production of the cement, expressed as follows:

(3) $$q_{c}\left(x,t\right)=C\cdot \frac{\partial Q}{\partial t}$$

The analytical expression of q_c_(x,t) and then Q(x,t) cannot be easily predicted as they depend on the actual temperature developed inside the concrete sample.

However, the following heat production function has been proposed in the case of adiabatic conditions and denoted as Q_a_(t) [20]:

(4) $$Q_{a}\left(t\right)=Q_{a,\max}^{*}\cdot e^{-{\left(\frac{\tau }{t}\right)}^{\beta }}$$

where the Q^*^_a,max_ (<Q_max_) is the actual amount of heat produced at the end of the reaction (in adiabatic conditions), whereas τ and β control the curve shape. Therefore, the rate of heat production q_a_(t) can be easily defined by introducing the expression of Q_a_(t) given by Equation (4) within the rate definition provided by Equation (3). However, the quantity q_c_(x,t) is generally not equal to q_a_(t) because different temperatures develop inside curing concrete under different boundary conditions. Nonetheless, an analytical relationship can be stated between q_c_(x,t) and q_a_(t) by considering the well-established Arrhenius principle [20], and then the former can be expressed in terms of the latter and substituted in Equation (2), which turns, then, into an integral-differential equation.

The initial and boundary conditions are needed to solve this problem. The initial condition can be generally written as follows:

(5) $$T\left(x,t=0\right)=T_{R}$$

where T_R_ is the initial room temperature (or ambient temperature). The boundary condition depends on the border between the curing concrete sample and the external environment.

In the case of non-adiabatic conditions, the following relationships can be imposed to guarantee the continuity of heat flow throughout the insulating layer and the external environment:

(6) $$\lambda_{p}\cdot \frac{T_{left}(t)-T_{R}}{t_{p}}=\lambda_{c}\cdot {\frac{\partial T}{\partial x}|}_{x=-L/2}\;\lambda_{p}\cdot \frac{T_{right}(t)-T_{R}}{t_{p}}=-\lambda_{c}\cdot {\frac{\partial T}{\partial x}|}_{x=L/2}$$

where L is the length of the specimen in the heat flow direction x, while t_p_ and λ_p_ are the thickness and the thermal conductivity of the insulating layer. Conversely, if the concrete is cured in “isothermal” boundary conditions at a room temperature T_R_ (supposed constant, for the sake of simplicity), the following boundary conditions should be considered:

(7) $$T\left(x=-L/2,t\right)=T_{R},\;T\left(x=L/2,t\right)=T_{R}$$

The solution of the integral-partial-differential Equation (2)**,** with the boundary conditions in Equations (6) or (7), can be employed to simulate the time evolution of T within the concrete element. The resulting theoretical simulation of the temperature field T_th_ can be symbolically denoted as follows:

(8) $$T_{th}=T_{th}\left(x,t;q_{r}\right)$$

where the vector **q**_r_ contains the following set of parameters listed below:

(9) $$q_{r}=\left[T_{R},Q_{\max},\alpha_{h,\max},c_{c},\rho_{c},\lambda_{c},\tau ,\beta {,\lambda }_{p}\right]$$

Regarding the components of the vector **q**_r_:

- the reference temperature (T_R_) can be assumed “a priori” according to the direct measurements;
- the “thermal” properties of the concrete (i.e., c_c_, ρ_c_ and λ_c_) are evaluated as a function of the mixture composition;
- the Q_max_ and α_h,max_ values can be determined by the analytical correlations available in the literature, as a function of the cement type and the water-to-cement ratio [21];
- the parameters τ and β are calibrated on adiabatic curves provided by recently released guidelines [22]: they depend on the cement type, as will be explained in Section 2.3;
- the thermal conductivity λ_p_ depends on the insulation properties of the materials used for covering the concrete mixture during the setting and hardening processes.

The values obtained for each mixture for **q**_r_ can then be utilized to simulate the actual hydration processes in the concrete elements. Details about the resolution of the integral-differential problem (i.e., solving Equation (2) with the boundary conditions of Equations (6) or (7)) are based on the procedure proposed by Martinelli et al. [17], in which the above-mentioned equation has been numerically solved and validated for the indirect identification of concrete adiabatic hydration curves based on semi-adiabatic temperature measurements [17].

### 2.2. Time Evolution of the Mechanical Properties

This section presents the simulation approach proposed for the assessment of the key mechanical performance of cement-based mixtures based on the hydration modelling procedure.

As a matter of principle, it is widely recognized that the degree of hydration α_h_ controls the key relevant mechanical properties of concrete during the hardening phase [18]. Specifically, for the sake of brevity, only the formulations for the main properties, i.e., the compressive strength (f_c_), Young modulus (E_c_) and tensile strength (f_ct_), are reported below [17]:

(10) $$\frac{f_{c}(\alpha_{h})}{f_{c}(\alpha_{h}=1)}={\left(\frac{\alpha_{h}-\alpha_{h,}{}_{0}}{1-\alpha_{o}}\right)}^{A}$$

(11) $$\frac{E_{c}(\alpha_{h})}{E_{c}(\alpha_{h}=1)}={\left(\frac{\alpha_{h}-\alpha_{h,}{}_{0}}{1-\alpha_{o}}\right)}^{B}$$

(12) $$\frac{f_{ct}(\alpha_{h})}{f_{ct}(\alpha_{h}=1)}={\left(\frac{\alpha_{h}-\alpha_{h,}{}_{0}}{1-\alpha_{o}}\right)}^{C}$$

where f_c_ (α_h_ = 1), E_c_ (α_h_ = 1) and f_ct_ (α_h_ = 1) represent the asymptotic theoretical maximum values for compressive strength, elastic modulus and tensile strength, in the ideal case of complete hydration (α_h_ = 1). On the other hand, α_0_ is the so-called critical hydration below which no relevant strength gain is achieved. The parameters A, B and C represent numerical values depending on the type of cement employed within the mixtures.

### 2.3. Input Data for the Parametric Analysis

The formulation of the numerical procedure outlined in Section 2.1 and Section 2.2 highlights that the hydration processes and, consequently, the time evolution of the mechanical properties of cement-based mixtures are influenced by the following parameters: (i) mixture composition (i.e., Q_max_, α_h,max_, τ and β); (ii) geometry of the concrete element (i.e., L); (iii) thermal insulation characteristics (i.e., t_p_ and λ_p_) during the setting and hardening phases; and (iv) the environmental conditions (i.e., T_R_).

In this context, a comprehensive parametric analysis is proposed in this study by considering the variation of each parameter. All the analyses proposed in the present research will be executed on three representative mixture compositions and, then, for each mixture, the other parameters (geometry, thermal insulation and environmental conditions) will be varied. Specifically, a variable mixture composition is realized by designing three concretes with the strength classes C25/30, C35/45 and C50/60 in accordance with the Eurocode 2 [23]. 

These mixtures are designed by keeping the water-to-cement ratio constant (i.e., equal to 0.40), as well as the maximum size of the aggregates (i.e., 16 mm) and the free water amount (i.e., 215 L/m^3^) in order to ensure also the desired workability (slump value ranging from 100 and 150 mm). Then, three different types of Portland cement, labelled CEM IV/B 32.5, CEM II/A-LL 42.5R and CEM I 52.5R in accordance with the EN 197-1: 2011 [24] are considered for achieving the three different characteristic strengths of the resulting mixtures at 28 days (i.e., R_ck_): 32 MPa for C25/30, 45 MPa for C35/45 and 60 MPa for C50/60 [23]. Table 1 summarizes the main characteristics of the concretes that will be considered for the parametric analysis proposed in the coming sections.

**Figure 2:**
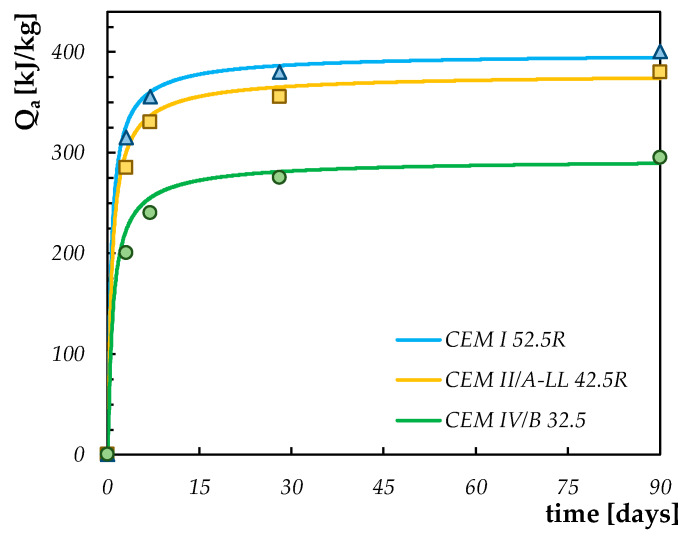
Calibration of hydration adiabatic curves for the cements considered in this study.

In accordance with the hydration model described in Section 2.1, the adiabatic curves for the different types of cements are evaluated by calibrating the parameters Q^*^_max_, τ and β defined in Equation (4). The calibration is based on the best fits of the values proposed by the Italian guidelines on structural concrete production [22]: the results of this calibration are proposed in Figure 2 beyond the points representing the values proposed by the Italian guidelines [22]. Moreover, the values obtained for the parameters defining the adiabatic curves are reported in Table 1.

On the other hand, in order to propose an assessment of the mechanical performance at the early-age, the numerical parameter presented in the Equations (10)–(12) should be defined.

Regarding the parameter α_0_, which represents the end of the setting phase and the start of hardening [21], in the literature, several values are proposed as a function of the concrete composition (e.g., cement type and water-to-cement ratio): generally, this parameter ranges between 0.17 and 0.40 [2]. Regarding the parameters A, B and C, respectively defined in Equations (10)–(12), in the literature several data are available. Specifically, for the time evolution of the compressive strength, De Schutter and Taerwe [25] consider a range of values for parameter A, moving from 0.84 (for CEM I) to 1.40 (for CEM III), meanwhile, parameter B (governing the time evolution of the elastic modulus) is equal to 0.26 and 0.62 for CEM I and CEM III, respectively. Moreover, other authors in the literature [26] assume that parameter B can be equal to one-third of the value defined for parameter A. 

Finally, for the tensile strength, parameter C assumes numerical values ranging from 0.46 and 0.88 [25].

**Figure 3:**
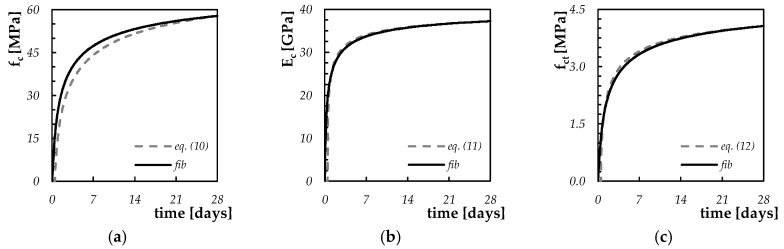
Comparison between the proposed equations and the *fib* Model Code 2010 formulas for the definition of the time evolution of the mechanical performances of C50/60: (**a**) compressive strength, (**b**) elastic modulus and (**c**) tensile strength.

In the present research, the aforementioned parameters α_0,_ A, B and C are calibrated (also based on the range of variation proposed in the literature) with the aim to obtain the best fit of the curves of the Equations (10–12), with the corresponding curves (i.e., time resistance) proposed by the *fib* Model Code 2010 [27]. In order to compare the formula proposed by the *fib* Model Code [27] with the relations reported in Section 2.2, for each mixture considered herein (i.e., C25/30, C35/45 and C50/60), numerical analyses for defining the time evolution of the mechanical properties as a function of the degree of hydration were performed on 15 cm concrete samples, in the absence of thermal insulation protection and an ambient temperature of 20 °C. The calibrated numerical values for the parameters α_0_, A, B and C are summarized in Table 1, while the “quality” of the calibrations are confirmed by comparing the curves obtained with the proposed model and the ones available from well-known structural concrete international guidelines (see the representative comparison for C50/60 proposed in Figure 3).

## 3. Temperature Profiles and Cracking Risk Assessment

**Figure 4:**
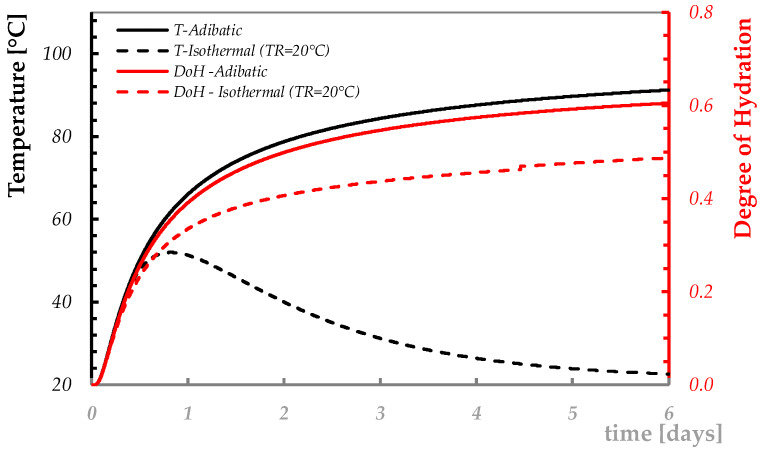
Representative simulation of the time evolution of temperature (principal vertical axes) and degree of hydration (secondary vertical axes) in adiabatic and isothermal conditions within a concrete element.

Once the concrete mixture’s composition is defined, the numerical procedure proposed in the previous section can be employed for simulating the time evolution of the temperature within a concrete element. For instance, Figure 4 shows the simulated temperature curve (see principal vertical axes in Figure 4) obtained in the middle point of a concrete element of 1 m thickness made from C50/60 concrete (Table 1) cured in an adiabatic calorimeter and when the concrete surface is not covered by any isolating layer and the ambient temperature presents a constant value equal to 20 °C (i.e., herein defined as the “isothermal boundary condition”). The figure shows that, despite the ambient temperature being constant, the thermal properties of the cement-based mixtures as well as the concrete element’s geometry do not allow the “free” heat flow, and this results in a “quasi-adiabatic” distribution of the temperature in the center of the 1-m thick element. Consequently, the time evolution of the degree of hydration is also influenced by the curing boundary condition, as highlighted in the secondary (red) vertical axes in Figure 4, showing the estimated evolution of the average degree of hydration over time within the 1-m thick concrete element, up to 6 days of curing.

**Figure 5:**
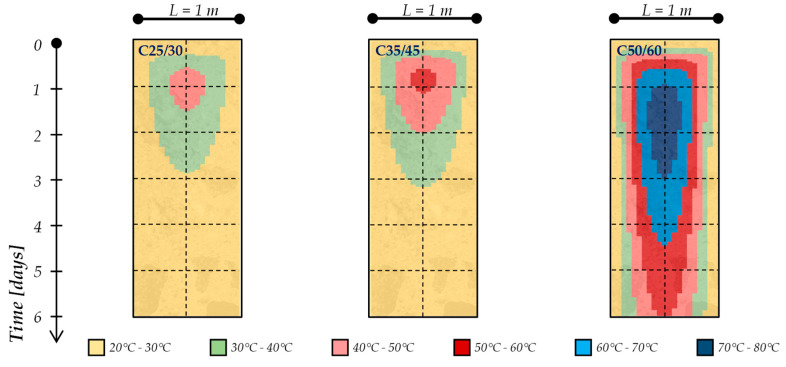
Time evolution of temperatures in the 1-m concrete elements during the hydration processes.

**Figure 6:**
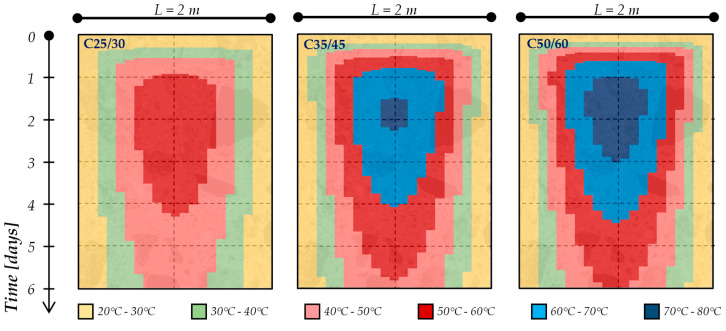
Time evolution of temperatures in the 2-m concrete elements during the hydration processes.

Moreover, these simulations lead to identifying the profile temperatures within the concrete elements during the setting and hardening phases. As a matter of fact, Figure 5 and Figure 6 present the temperature profiles within a 1-m and 2-m thick concrete element, respectively, for the mixtures considered in the present study (i.e., C25/30, C35/45 and C50/60), cured in an isothermal condition with an ambient temperature equal to 20 °C. Specifically, the colored maps reported in Figure 5 and Figure 6 show the time (vertical axis, up to 6 days) and space (horizontal axis, representing the element’s thickness) temperature variation within the concrete element.

The figures highlight that, already during the first 48 h, the cement hydration lead to the achievement of the maximum temperature rising (T_max_) in the center of the concrete element as well as the maximum gradient of temperature between the several layers of the element (ΔT_max_): these values, as expected (due to the hydration kinetics), are more significant for the C50/60 mixture (containing CEM I 52.2R) and this is more pronounced when the concrete element’s geometry increases (i.e., moving from 1 m to 2 m thickness for all types of mixtures).

These data allow one to perform an “indirect” control of the cracking risk for cement-based mixtures. This procedure is based on the recent guidelines released in Italy by the Superior Council of Public Works [22]. In fact, in accordance with the above-mentioned guidelines, in order to prevent the cracking risk due to the thermal stresses, the following limits should be verified in concrete elements during the setting and hardening phases:

- Maximum concrete temperature ≤ 70 °C;
- ΔT_max_ ≤ 20 °C between the various parts of the structure.

Therefore, as the model demonstrates, to have the ability to capture the evolution of temperature during the cement hydration, the following subsections show the results of a comprehensive parametric analysis intended to show the influence of the relevant parameters, such as the geometry of the concrete element and the concrete curing conditions (i.e., thermal insulation characteristics and/or external ambient temperature), on the cracking risk occurring in structural concrete elements during the setting and hardening phases.

### 3.1. Influence of Concrete Element Geometry

The geometry of the structural element plays a fundamental role on the temperature developed inside the mixture due to the cement hydration. For instance, in the case of massive structures, such as slab foundations of multi-storey buildings and retaining walls of considerable height, due to the low thermal conductivity of the concrete, the external layers of the structural elements prevent the dissipation of the generated heat during the hydration from the center towards the outside, causing a significant increase in the temperature. The cortical layers settle on lower temperatures than those that occur in the heart of the structure, giving rise to a thermal gradient, which can determine the appearance of the critical crack patterns.

For the parametric analysis, the following input parameters were considered for simulating the concrete behavior of the mixture considered in this research:

- Ambient temperature set to 20 °C;
- Absence of thermal insulation protection;
- Variable thickness between 0.50 and 1.50 m.

**Figure 7:**
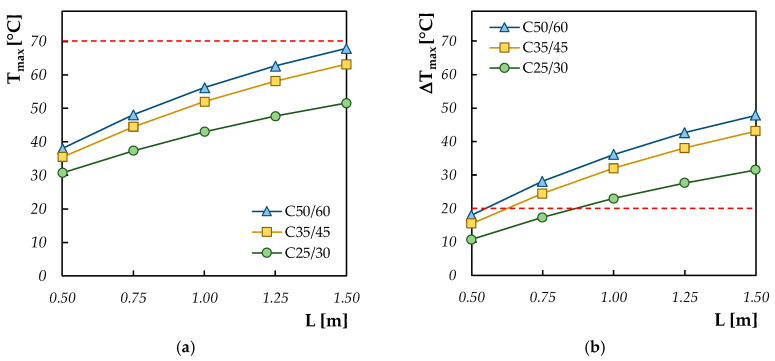
Maximum temperature (**a**) and maximum thermal gradient (**b**) by varying the concrete element’s thickness.

Figure 7 summarizes the obtained result. It should be noticed that, while the maximum temperature in isothermal conditions never exceeds the limit recommended by the guidelines, the thermal gradient between the core and periphery of the hardening mixture exceeds this limit already for a thickness of the element of about 80, 60 and 55 cm for C25/30, C35/45 and C50/60, respectively. Moreover, as expected, by increasing the thickness of the structural element, both the maximum temperature registered within the center of the element and the temperature gradient generated along the element profiles increase, leading to facilitate the formation of thermal cracks.

### 3.2. Influence of Thermal Isolation of the Formwork

Further, in the light of the results obtained in the previous subsection, it is clear that the attenuation of the thermal gradient in massive structures can be pursued through a reduction of the gap between the maximum temperature achieved within the core of the element and the outer layers of the elements that are in direct contact with the external ambient: this could be also obtained by adopting a protection of the outer layers through an adequate thermal insulation system. As a matter of fact, by adopting insulation systems, the dissipation of heat towards the environment is limited, increasing the temperature of the heart but significantly decreasing the thermal gradient, which causes cracking.

For the analysis proposed hereafter, the following parameters are considered as “invariant”: an ambient temperature of 20 °C and a thickness of 1 m, which, as seen in the previous subsection, in the absence of insulation system, exceeded the limit value recommended by the guidelines to prevent thermal cracking. In particular, the key goal is to observe the changes in terms of the maximum thermal gradient and maximum temperature, considering the following three cases:

(i) Absence of thermal insulation (i.e., λ_p_ = ∞);
(ii) Intermediate thermal insulation system (i.e., λ_p_ = 0.125 W/mK) realized with wooden formwork;
(iii) High thermal insulation system (i.e., λ_p_ = 0.065 W/mK) realized with fir-mineralized wood bonded with Portland cement formworks. For both the thermal insulation systems, an equal thickness of the formwork’s panel (i.e., 2 cm) is considered.

**Figure 8:**
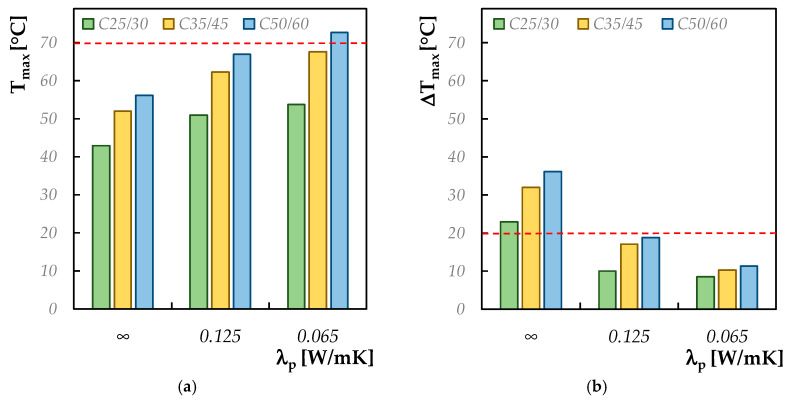
Maximum temperature (**a**) and maximum thermal gradient (**b**) by varying the formwork typology.

The results of the present parametric analysis are summarized in Figure 8. It highlights that the thermal protection of concrete during setting and hardening, already with a 2 cm wooden formwork, allows one to mitigate the risk of thermal cracking.

On the other hand, a higher maximum temperature is reached within the core of the element as the thermal insulation system is improved: this leads to exceeding the limit recommended by the legislation when the concrete thicknesses increase and/or when a higher class of cements are employed (i.e., moving from C25/30 to C35/45 to C50/60). Conversely, the thermal gradient between the core and the cortical layers is reduced. Therefore, the application of a thermal insulation system for the protection of the cast mixture leads to mitigate the thermal cracking risk only in some cases and for certain thicknesses and types of cements.

### 3.3. Influence of Ambient Temperature

In addition to the measures aimed at reducing the development of the hydration heat that directly affects the concrete components or the technological aspects, another factor that also has a key influence on the formation of thermal cracks is the environmental condition.

**Figure 9:**
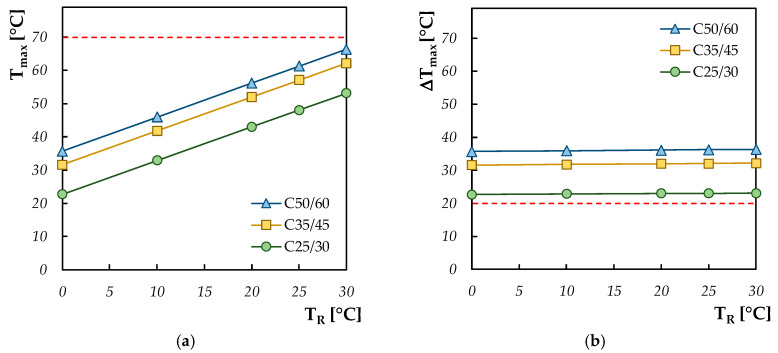
Maximum temperature (**a**) and maximum thermal gradient (**b**) by varying the ambient temperature.

Therefore, in this subsection, a parametric analysis is conducted considering the variation of the ambient temperature, going from cold (T_R_ = 0 °C) to hot (T_R_ = 35 °C) climates. Further, in this case, the other input parameters are considered as “invariant”: element’s thickness of 1 m and absence of thermal insulation protection. The results of the simulations are reported in Figure 9. It can be observed that the maximum temperature reached inside the concrete element is directly influenced by the ambient temperature, while the thermal gradient that develops between the heart and the periphery remains almost constant.

To reduce the inside temperature which exceeds, in the case of a hot ambient temperature, the limit set by the Italian guidelines [22], it is possible to reduce the temperature of the fresh concrete mixture during casting, for example, by cooling down its ingredients [2].

## 4. Mechanical Properties at Early-Age

The approach proposed in the previous sections allows one to perform an “implicit” verification of the thermal crack formations in accordance with and based on the development of the temperature profiles within the concrete elements. On the other hand, in the view of developing the present model by “explicitly” verifying the thermal cracking risk, the first step is represented by the estimation of the mechanical properties’ strength development (in time and along the concrete thickness): these values could be then compared with the thermal stresses generated during the hydration.

**Figure 10:**
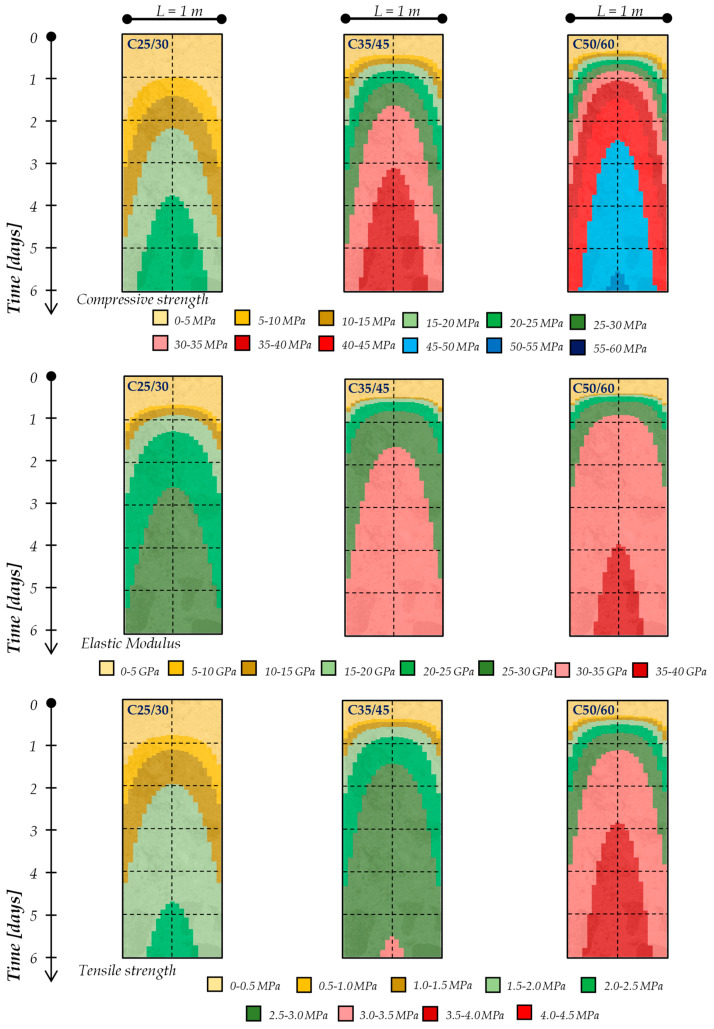
Early-age mechanical properties of 1-m thick concrete elements.

**Figure 11:**
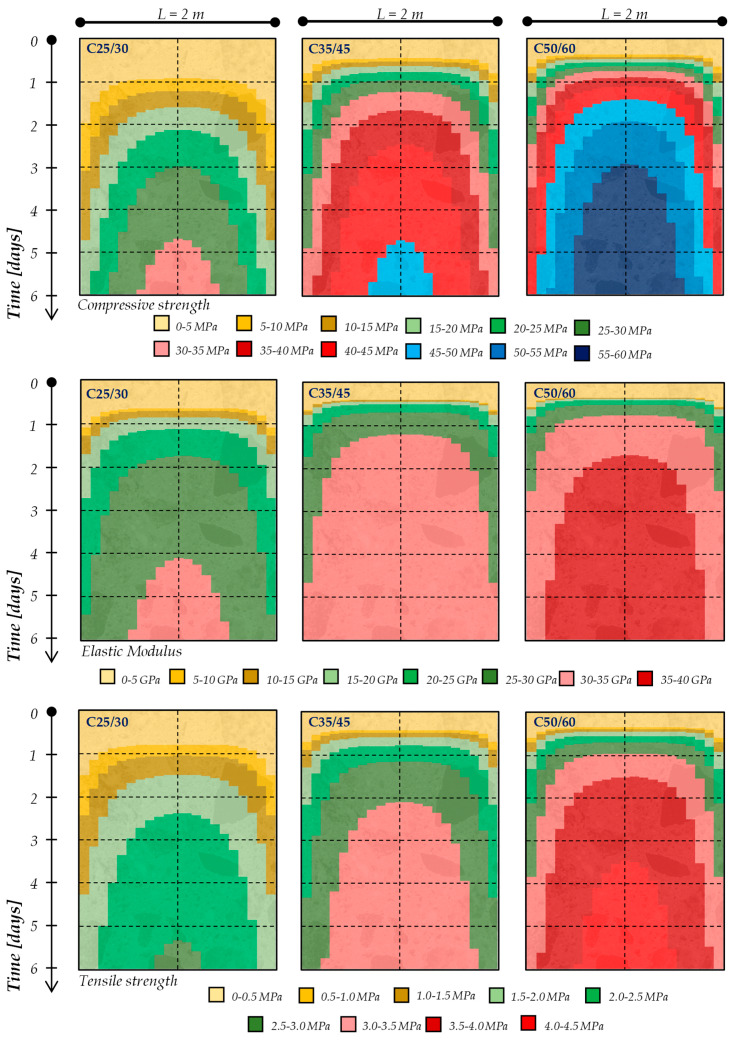
Early-age mechanical properties of 2-m thick concrete elements.

In this context, Figure 10 and Figure 11 show the time and space evolution of the compressive strength, elastic modulus and tensile strength by considering the variation in the concrete strength class and the thickness. Specifically, the colored maps reported in Figure 10 and Figure 11 show the time (vertical axis, up to 6 days) and space (horizontal axis, representing the element’s thickness) mechanical properties’ variation within the concrete element.

It can be seen, therefore, that the “grid” of the degree of hydration (i.e., its development in time and space) corresponds to the “grid” of the key mechanical performances by considering Equations (10)–(12) defined in Section 2.3. Consequently, as occurs for the degree of hydration, despite the curing boundary condition being kept constant, the thermal properties of the cement-based mixtures as well as the element’s geometry do not allow the “free” heat flow, and this results in a “quasi-adiabatic” distribution of the degree of hydration; consequently, the time and space evolution of the mechanical performances are directly influenced.

In fact, the results reported in both Figure 10 and Figure 11 highlight that strength does not evolve homogeneously throughout the element thickness, as the external layers develop the resistance more slowly than the internal ones: this is due to the higher temperature (see also Figure 5 and Figure 6) achieved in the core of the element. Therefore, the thermal gradient between the heart and periphery is also transformed into a difference in resistance reached by the various parts.

Then, for a given concrete mixture, it is noted that, as the thickness increases, in the center of the element, the temperature increases (Figure 5 and Figure 6) and consequently, the evolution of the mechanical properties continually speeds up (Figure 10 and Figure 11). In addition, since the cortical layers mature at the ambient temperature, by increasing the temperature in the core of the casting, the thermal gradient also increases, and therefore the non-homogeneity in the development of the mechanical properties is more pronounced. It is worth highlighting that these observations, are more significant when a higher cement strength class is used (i.e., moving from C25/30 to C35/45 to C50/60). It is also worth highlighting that the early-age mechanical properties’ development is faster for the elastic modulus and lower for the tensile strength.

Finally, although, for the sake of brevity, all the parametric analysis figures are not reported herein, the simulations highlight that the mechanical properties are strictly related to the temperature and consequently, their development is faster when the ambient temperature increases and/or when thermal insulation systems are employed for protecting the mixture curing.

## 5. Conclusions

This study presented a numerical procedure for simulating the effects of generated hydration heat in hardening concrete. Considering the variation of the several factors, the simulations lead to propose a simplified procedure for mitigating the cracking phenomena. The following conclusions deserve to be highlighted:

- the proposed model is capable to simulate the time evolution of both the temperature and degree of hydration in concrete during the setting and hardening phases of the hydration processes;
- the proposed 1-D flow and hydration model turns out to be a useful tool for assessing the cracking risk due to thermal stresses;
- the parametric analysis proposed herein allows to quantify the influence of key parameters affecting the thermal crack formations, such as the geometry of the element, the type of formwork employed for the thermal protection and the ambient temperature;
- the figures derived from the parametric analysis can be employed as simplified design abaci for the optimization of both concrete mixtures as well as the construction phases aimed at mitigating the thermal cracking risk: similar simulations can be performed for other types of mixtures leading to a more comprehensive set of data that can be used for practical aspects;
- the model can be easily employed also for estimating the time evolution of mechanical properties such as the compressive strength, the tensile strength and the elastic modulus: this represents the first step toward a more advanced simulation tool for the “direct” verification of thermal crack formations.

Finally, as a further step of the present research, the proposed approach can be applied in the case of relevant case studies and, moreover, the numerical procedure can be implemented also to the 3-D domain in order to be proposed for a wide range of possible applications.

## Figures and Tables

**Table 1:** Concrete mixture characteristics.

Description	C25/30	C35/45	C50/60
Cement type	CEM IV/B 32.5	CEM II/A-LL 42.5R	CEM I 52.5R
R_ck,28_ [MPa]	32.00	45.00	60.00
f_cm,28_ [MPa]	34.56	45.35	57.80
Q^*^_max_ [kJ/kg]	295	380	400
τ [h]	15.0	12.0	10.0
β	0.80	0.80	0.80
α_0_	0.25	0.20	0.20
A	1.40	0.86	0.84
B	0.47	0.29	0.28
C	0.88	0.55	0.55
